# A controlled aquarium system and approach to study the role of sponge-bacteria interactions using *Aplysilla rosea* and *Vibrio natriegens*

**DOI:** 10.1038/s41598-018-30295-y

**Published:** 2018-08-07

**Authors:** Mohammad F. Mehbub, Jason E. Tanner, Stephen J. Barnett, Jan Bekker, Christopher M. M. Franco, Wei Zhang

**Affiliations:** 1 Centre for Marine Bioproducts Development, College of Medicine and Public Health, Flinders University, Bedford Park, SA 5042 Adelaide, Australia; 2 Medical Biotechnology, College of Medicine and Public Health, Flinders University, Bedford Park, SA 5042 Adelaide, Australia; 3 SARDI Aquatic Sciences, 2 Hamra Avenue, West Beach, SA 5024 Adelaide, Australia

## Abstract

Sponge-bacteria interactions are very important due to their ecological and biological significance. To understand the impact of interactions between sponges and bacteria (both associated with and external to sponges) on sponge-associated microbial diversity, sponge metabolite profiles and bioactivity, we used a controlled aquarium system and designed an experimental approach that allows the study of sponge-bacteria interactions in a well-defined manner. To test the feasibility of this approach, this system was used to study the interaction between a sponge *Aplysilla rosea* and a marine bacterium commonly found in seawater, *Vibrio natriegens*. Sponge explants were exposed to *V*. *natriegens*, at 5 × 10^6^ cfu/ml, and changes were monitored for 48 hours. Pyro-sequencing revealed significant shifts in microbial communities associated with the sponges after 24 to 48 hours. Both the control (sponge only without added bacteria) and *Vibrio*-exposed sponges showed a distinct shift in bacterial diversity and abundance with time. *Vibrio* exposure significantly increased bacterial diversity, the abundance of a number of taxa compared to control sponges. The result experimentally supports the notion of dynamic and concerted responses by the sponge when interacting with a bacterium, and demonstrates the feasibility of using this controlled aquarium system for the study of sponge-bacteria interactions.

## Introduction

Sponges (phylum Porifera) host a distinct and diverse microbial community of symbiotic bacteria, archaea and unicellular eukaryotes^1^. Being sessile filter-feeders, any microorganisms ingested by a sponge that can resist its digestive processes and immune response may successfully maintain a symbiotic relationship with the host^2,3^. However, sponges do have the ability to distinguish between bacteria that they filter out of the water column and their own symbionts^4^. Bacteria can inhabit sponges both intracellularly and extracellularly^5,6^, and the resultant interactions are very important for sponge survival, and maintenance of their ecology and biology^1^.

Chemical defence is frequently used by sponges to protect themselves from predation^7^, microbial infections, biofouling, and overgrowth by other sessile organisms^8^. Sponges possibly also utilize their symbionts as a defence mechanism against pathogens by stimulating them to produce chemicals that inhibit the colonization of potential pathogenic bacteria^9,10^. Sponge-bacteria symbioses^11^ have been widely studied in order to gain an understanding of the biosynthetic origin of bioactive secondary metabolites^12^.

Over 30% of all the marine natural products discovered so far are derived from sponges, making them the greatest producers of bioactive metabolites among marine organisms^13,14^. These natural products, such as smenamides, dolastatins and curacins^15–17^, possess great potential for drug development. An understanding of the ecology and functional diversity of marine symbionts associated with sponges and how they interact to produce bioactive metabolites could lead to the development of production strategies for the drug leads from sponge sources^18–20^.

While interactions between sponges and associated bacteria are of significant interest^21^, limited studies have been conducted to understand their impacts on microbial diversity, metabolite profiles, and bioactivity following exposure to foreign bacteria^22^. In our previous study, the technical feasibility of such an approach was tested by challenging the sponge *Aplysilla rosea* with its associated bacterium *Streptomyces* ACT-52A in a controlled aquarium^22^. It was found that the interaction between the sponge and *Streptomyces* ACT-52A played an important role in regulating secondary metabolite production. However, this approach has not been used to understand the interactions between sponges and external foreign bacteria, such as the common marine bacterium *Vibrio natriegens*^23^ used in this study. Therefore, the goal of this study is to test the feasibility of this approach by studying the interaction between the sponge *Aplysilla rosea* and the external foreign bacterium, *Vibrio natriegens*.

Although the microbial communities of most sponges stay very stable under environmental changes^24^, some show substantial changes when exposed to an environmental change^25–27^. Based on our recent study^22^, we hypothesized that the interactions between a sponge and the external foreign bacteria in its surrounding environment, could play an important and different role as compared to its associated bacteria in changing the sponge microbial diversity, morphology and bioactivity. We chose *Vibrio* as aquatic environments are a rich source of various *Vibrio* spp.^28,29^ that can survive in a range of environmental conditions and in eukaryotic hosts such as, sponges^30,31^. *Vibrio natriegens* was originally isolated from salt marsh mud^32^. We selected this bacterium because it has not been identified as associated with *A*. *rosea*, grows in the marine environment^23^, and has a very short generation time of less than 10 min under optimal growth conditions^33^.

## Materials and Methods

### Sponge collection, transport and laboratory maintenance

Twelve large pieces (>10 cm) of the shallow water sponge *A*. *rosea* were collected from Rapid Bay, Gulf St Vincent (35°31′15.29′′S, 138°11′6.88′′E) in South Australia at a depth of 9 m, in October 2011. Sponges were carefully removed from jetty pylons with a scraper, kept immersed in seawater in plastic bags, and immediately transferred to buckets of seawater on the surface without exposure to air. Sponges were transported to the laboratory with aeration and held in the transport buckets through 5–6 water changes at 20-minute intervals to remove any toxins released as a result of transport stress. Next, they were transferred into aquaria (100 L capacity) for 1–2 weeks with non-sterile natural seawater exchange on alternate days. Sponges were fed with 40 ml of the microalga *Nanochloropsis* sp. at 10^6^ cfu/ml every 2 to 3 days. Feeding was stopped 24 hours prior to start of the experiment.

### Media preparation, culture of *Vibrio natriegens* and cell count

*Vibrio natriegens* can grow, in a wide range of media. However, Luria agar (LA) and Luria broth (LB)^34^ was selected as standardized growth media due to the simplicity and accessibility of its formulation^35^. LA and LB, were prepared to grow *V*. *natriegens* (kindly provided by Lyn Spencer, Biological Science from Flinders University) with added tryptone, 10 g/L; yeast extract, 5 g/L; NaCl, 22.5 g/L; with and without 15 g/L agar^3^. A liquid culture of *V*. *natriegens* was prepared by inoculating cells from a single colony on LA medium into 100 ml LB medium and incubated at 37 °C for 12 h on a rotary shaker at 200 rpm. Cultures were centrifuged to remove the growth medium, re-suspended in sterile seawater and added to tanks to a final concentration of 5 × 10^6^ cfu/ml at the start of the experiment (Time 0 h).

Cell counts of *V*. *natriegens* from seawater were conducted by dilution series and plating onto LA media using the Miles and Misra method^36^. Briefly, two replicates of a 1 in 10 serial dilution series of 10^−1^ to 10^−12^ were prepared in 900 μl of 0.9% sterile saline. Absorbance was measured at 550 nm for each dilution using a UV-Vis spectrophotometer, and a standard curve was prepared to correlate the absorbance to cell concentration. *V*. *natriegens* was readily identified by comparison of colony morphology with the pure culture.

### Experimental design

Several pilot experiments were conducted with the following aquarium set-up to ensure the consistency of the system. An aquarium of 10 L capacity was used with 5 L of sand filtered seawater for the final experiment. Three replicate aquaria were used for each of the three treatments; sponge only control (C), bacteria only control (BC) and the sponge plus *V*. *natriegens* treatment (T). Fine bubble aeration was provided to maintain dissolved oxygen levels at 6–10 ppm. Sponge explants (n = 36) were cut from 9 (nine) large sponges (1 sponge = 4 pieces) with a sterilized sharp knife under seawater, and averaged 6.02 ± 1.11 g wet weight and ca. 6 cm in diameter. Sponge explants were maintained in six 100 L aquaria for 4–5 days prior to conducting the experiment to allow acclimatization. Six sponge explants were added to each of the three replicate sponge only control and treatment aquaria. The replicate sponge only control and treatment tanks contained at least one sponge explant from the same larger sponge for better comparison. A 12 hr day/night cycle was maintained during the experiment.

For sampling, one sponge explant was collected at 0, 6, 12, 24 and 48 hours from each tank, placed in a sterile 50 ml tube, frozen immediately in liquid nitrogen, freeze-dried then ground with a sterile mortar and pestle into fine powder and stored at −80 °C. Seawater in each tank was monitored for bacterial cell count, pH, ammonia, nitrate, nitrite (by a commercial kit Aqua One, Kong’s Pty Ltd, Australia), and absorbance at 0, 3, 6, 12, 24 and 48 hours at 550 nm wavelength.

Samples were labeled with the following formats: sponge with *Vibrio* treatment (T); sponge only without *Vibrio* treatment as Control (C) and *Vibrio* bacteria only control without sponge (BC), followed by time of sampling in hours and replicate tank number. For example, C0–1 means sponge only control at 0 hour, replicate 1; T48–3 means *V*. *natriegens* treated sponge at 48 hours, replicate 3.

### Genomic DNA extraction and analysis

To assess the microbial community associated with each sponge explant, we used 454 pyrosequencing. Briefly, total genomic DNA was extracted from 20 mg of lyophilized sponge tissue using a modified DNA extraction method^37^. The main modification was in the buffer composition (3% CTAB, 100 mM Tris-HCl, 2 M NaCl, 20 mM Na_2_EDTA, 0.2% LiCl, 2% PVP and 1% *β*-*mercaptoethanol*), which, along with another buffer (lysozyme 80 mg/mL, 4.8% Triton X, 8 mM EDTA and 80 mM Tris-Cl) and a tissue lyser (Qiagen), was used for mechanical disruption with 1 mm beads for 30 sec at 15 Hz speed. Two separate extractions from the same sample were combined. DNA was purified using DNeasy Blood and Tissue Kits (Qiagen) and quantified in a ND-8000 UV-Visible Spectrophotometer (Thermo Scientific). The 16 S rRNA genes were amplified for 454 pyrosequencing from 50 ng of DNA using pyro 28 F and pyro519R primers^38,39^. A Hot Start Taq Plus master mix kit (Qiagen, Valencia, CA) was used for PCR under the following conditions: initial denaturing at 95 °C for 5 min, followed by 35 cycles of denaturing at 95 °C for 30 s, annealing at 54 °C for 40 s, and extension at 72 °C for 1 min, finalized by a 10-min elongation at 72 °C. Resulting PCR products were purified using Diffinity Rapid Tip (Diffinity Genomics, Inc., West Henrietta, NY) chemistry and replicate reactions pooled. Small fragments were removed with Agencourt AMPure Beads (Beckman Coulter, Brea, CA).

Tag-encoded FLX amplicons were sequenced on the Roche 454 FLX sequencer using Titanium (Roche) reagents. Multiplex raw sff files were analysed using a hybrid analysis pipeline. In brief, denoising was performed using a custom pipeline composed of Q25 trimming of all reads, clustering using USEARCH at 96% to remove singletons, de novo chimera identification using UCHIME on the clusters, followed by per-base error correction on the remaining non-chimeric sequences. The denoised sequences were then quality checked and any reads not matching a barcode and not at least 250 bp were removed. The reads were then dereplicated using USEARCH, and the resulting cluster reads were classified using BLASTn + against a custom database of GenBank 16 S sequences identified at least to the genus level. Sequences with at least 75% query coverage were classified to the species level if they were at least at 97% identity, genus if at least 95% identity, family if at least 90%. Classifications were compiled at each taxonomic level and ambiguities were identified when multiple assignments were possible. The full part of pyrosequencing and bioinformatics pipeline conducted by the Research and Testing Laboratory located in USA which is available at: http://www.researchandtesting.com/docs/Data_Analysis_Methodology.pdf.

### Metabolite profiling by high performance liquid chromatography (HPLC)

To assess sponge metabolite profiles, HPLC analyses were performed with an Agilent 1100 system, including Alliance separation module 2695, column heater, and 2998 photodiode array detector, and run using Empower Chromatography Data Software (Waters). The HPLC conditions consisted of two eluents (eluent A [0.1% aqueous trifluoroacetic acid] and eluent B [100% acetonitrile]) and an elution profile based on a linear gradient from 10% eluent B to 100% eluent B within 25 min and then holding at 100% eluent B for an additional 20 min. Flow rate was kept constant at 1 ml/min. Chromatographic separation was performed on an Atlantis T3 C18 column (Agilent, 100 mm × 3 mm ID and 3 µm particle size) in reversed phase with a fixed temperature of 25 °C. For the profiling of the polar metabolites, 100 mg of freeze-dried sponge tissue was extracted three times according to Turon *et al*.^40^. Briefly, powdered sponge tissue was transferred to a new safe lock tube (B147671L/2423, Eppendorf AG, Germany) and dissolved with 1 ml methanol in an ultrasonic tank for 5 min with high energy setting, centrifuged and the pellet retained after transferring the supernatant. The pellet was extracted twice more at 10 and 15 min sonication. The combined crude extracts were concentrated by using a centrifugal evaporator and finally dissolved with 1 ml methanol. This crude extract was filtered through a 13 mm 0.2 µm PVDF acrodisc Syringe Filter and added to a 2 ml tube with glass insert. Then, 50 µl of this filtered solution was injected into the HPLC system described above. The peaks were detected at 215, 250, 310, 365 nm from the data collected across the 200 to 800 nm wavelength range.

### Thin layer chromatography (TLC)

To further elaborate the nature of the metabolites produced, we used TLC. Samples were prepared in a similar way as for HPLC and 20 µl loaded onto aluminium TLC silica gel 60 F_254_ plates (Merck), using the following solvent systems: Ethyl acetate: Methanol [7:3] and Butanol: Acetic acid: water [4:1:1]. Preparative scale layer chromatography (PLC) was carried out using PLC glass silica gel (Kieselgel 60 F_254_, size 5 × 10 cm), with the active extracts against *Stayphyllococcus aureus* purified to obtain compounds for further analysis.

### Antibacterial activity test and bioautogram

To assess the antibacterial activity and to identify the active metabolites, *Staphyllococcus aureus* and *Escherichia coli* were grown on Tryptone soy agar (TSA, Tryptone soy 30 g, agar 15 g and reverse osmosis (RO) water 1 L)^41^ and then added to Antibiotic agar medium (AAM) (Sigma-Aldrich) at 1% (v/v, OD of 0.2 at 600 nm) prior to pouring plates. Crude sponge extracts (50 ul) were prepared in a similar way as for HPLC and added to wells in the agar. Pure methanol (without any sponge extracts) used as a control. Plates were incubated at 37 °C for 16 to 24 hours, and zones of inhibition were measured. *Vibrio natriegens* was grown in *Vibrio* agar (Brain heart infusion agar 38 g, NaCl, 20 g and MilliQ water, 1 L) and the antibacterial test was conducted in the same manner as described above except *Vibrio* agar was used instead of AAM.

### Statistical analysis

The percentage of each microbial species was used for hierarchical cluster analysis, and analysis of variance (ANOVA) and permutational multivariate analysis of variance (PERMANOVA) were used to determine differences in abundance of individual microbial taxa and composition respectively between treatments and sampling times. Species of *Endozoicomonas*, *Ruegeria*, *Tenacibaculum* and *Vibrio* (except for *V*. *natriegens*) were combined at the genus level, as the percentage of individual species was low (generally <1%). The microbial diversity in each sample was measured using Shannon’s Diversity Index (H′), with differences due to time and *Vibrio* addition analysed using ANOVA with treatment and time as orthogonal factors, and tank nested within treatment. GenStat version 16.1 was used for ANOVA analysis and post hoc comparison of differences were conducted using Fisher’s least significance difference (LSD). PERMANOVA was undertaken in the Primer v6 software to reveal the effects of experimental treatments on the composition of the entire microbial community^42^, using fourth root transformed data, Bray-Curtis similarities and 4999 permutations of residuals under a reduced model.

HPLC chromatograms were analysed with Empower Chromatography Data Software (Waters). Partial least squares-discriminant analysis (PLS-DA) was conducted using the chemometrics and mixOmics packages for R^43,44^ to compare the metabolite profiles from the control and treated sponge extracts at different time points. Antibacterial activity data were evaluated by Welch two sample t-test with two independent groups using R version 3.3.3.

### Sequence accession numbers

All sequence data were deposited, with MIMARKS-compliant metadata, in the NCBI Sequence Read Archive under Bio Project number PRJNA276919, with accession numbers from SRX950227 to SRX950240 (Supplementary File A).

## Results

### Laboratory maintenance of the sponge *Aplysilla rosea*

Large pieces (>10 cm) of *A*. *rosea* were successfully maintained in recirculating aquarium systems (100 L) for up to 40 days with feeding and regular seawater exchange. In the small experimental tanks (10 L), 5–6 small pieces (≤6 cm of length) were able to survive for a maximum of 10 days without feeding, and with added *Vibrio* at 10^6^ cfu/ml, they were able to survive for a maximum of 54 hours before they started to decay. Therefore, the experiments were restricted to 48 hours. Sponge health status was assessed visually by colour, tissue compactness and the appearance of necrotic spots. Supplementary File B shows sponge explants with and without added *Vibrio* at different time points as well as after 54 hours.

### Enumeration of total *Vibrio natriegens* cell count

**Figure 1:**
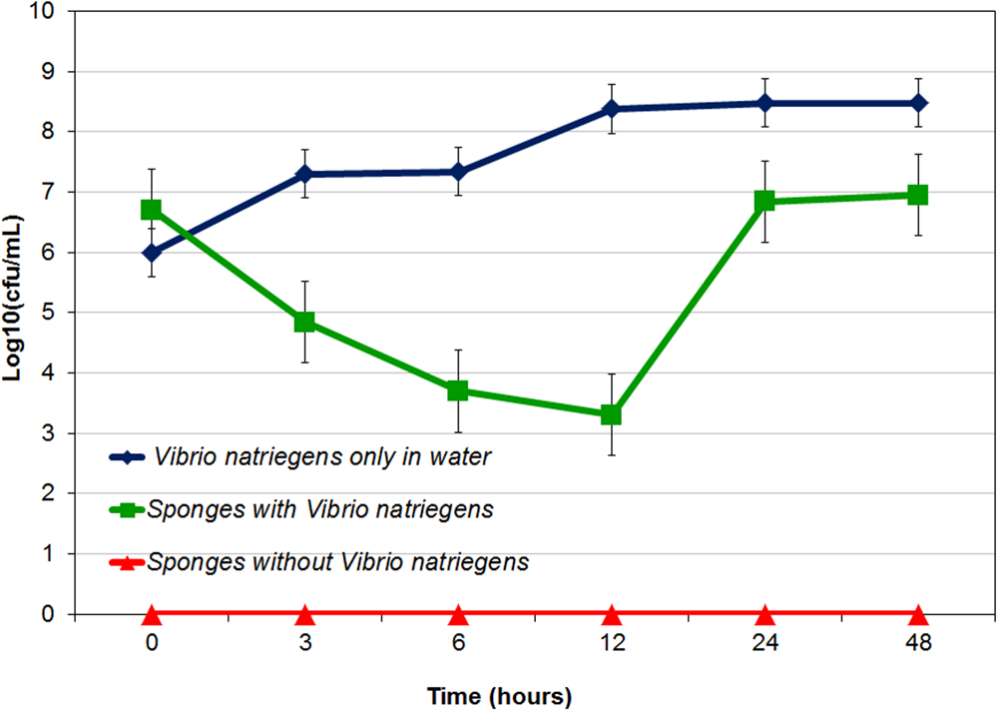
CFU counts of *Vibrio natriegens* in tank water over 48 hours after *V*. *natriegens* addition. Error bars indicate SEM, n = 3.

In control tanks without sponges (BC), the *Vibrio* culture grew rapidly and increased to 3 × 10^8^ cfu/ml at 24 hours and remained at this level until 48 hours (Fig. 1). In treatment tanks that contained sponges, the concentration of *V*. *natriegens* declined to 2 × 10^3^ cfu/ml at 12 hours, followed by an increase to 9 × 10^6^ cfu/ml at 24 hours (Fig. 1). *V*. *natriegens* cells were not detected in tanks containing only sponges.

### Characterization of microbial diversity by 454 pyrosequencing

**Figure 2:**
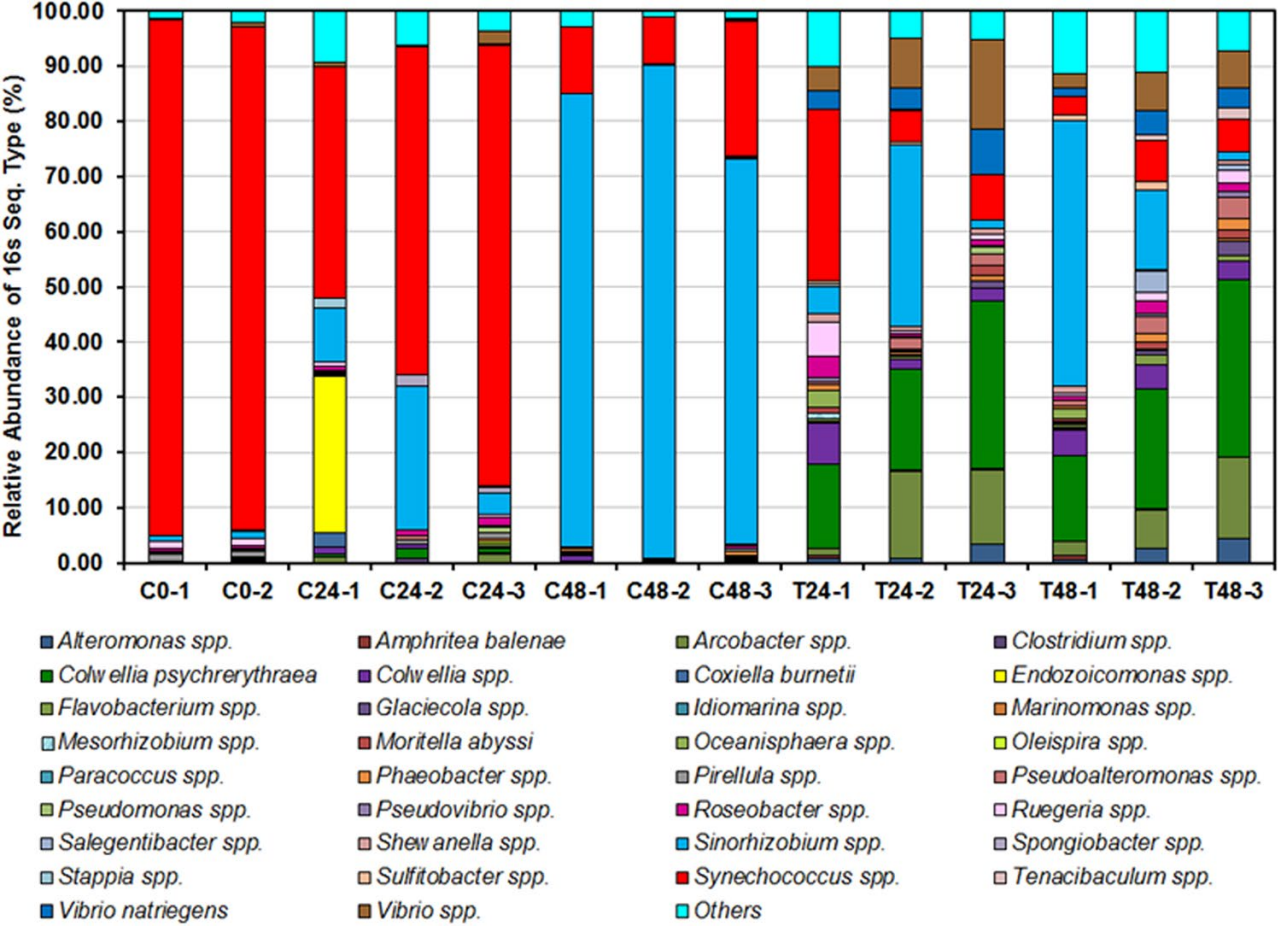
Different bacterial genera inferred from 16S rRNA gene relative sequence abundances derived from 454 pyrosequencing of sponges from control tanks (C, no added bacteria) and treated tanks (T, added *V*. *natriegens*) at 0, 24 and 48 h for each replicate tank.

**Figure 3:**
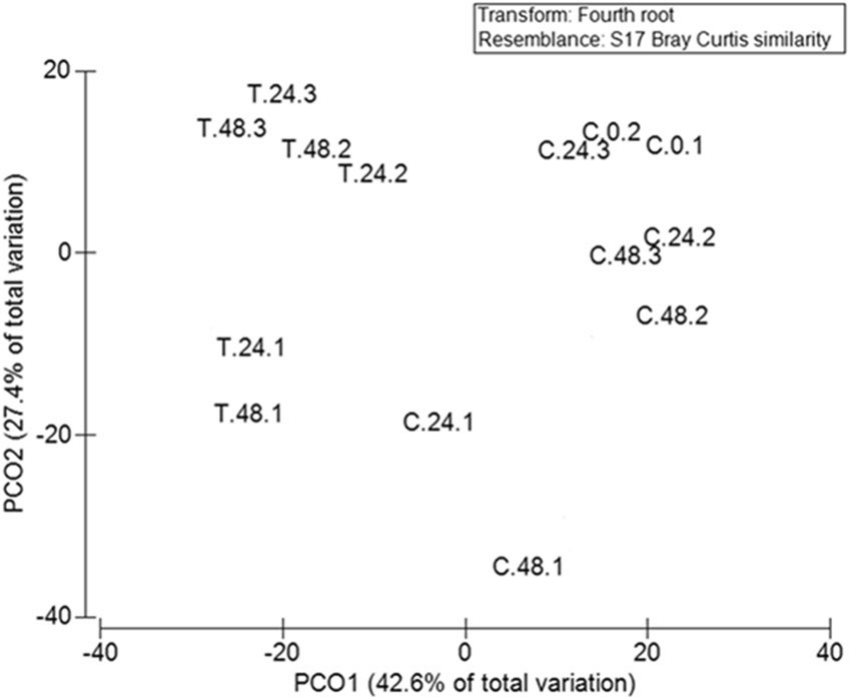
Principle Coordinate Ordination (PCO) of the bacterial communities associated with treated and control *Aplysilla rosea* sponges based on Bray Curtis similarity using 454 pyrosequencing of 16S rRNA gene.

Pyrosequencing revealed significant changes in the sponge-associated microbial communities, PERMANOVA indicated that both time and *Vibrio* treatment changed microbial community structure, although there was no interaction between the two (Table 1). Microbial diversity, as assessed with the Shannon Diversity index (H′), increased over time (ANOVA: F_2,9_ = 17.06, *P* = 0.001, H′ = 0.38, 1.52 and 1.24 for T = 0, 24 and 48 hours, respectively), pairwise tests indicate 0 hr < 24 hr = 48 hr, and was significantly (F_1,9_ = 67.18, *P* = 0.001) more diverse in the *Vibrio*-treated sponges (*H* = 2.07) compared to the control sponges (*H* = 0.80). There was no interaction between time and *Vibrio* treatment (F_1,9_ = 3.34, *P* = 0.101). In the control tanks, the sponge-associated bacterial community was initially dominated by *Synechococcus*, which decreased with time and was replaced by *Sinorhizobium* (Fig. 2). In the treated tanks, Principal coordinate analysis ordination (PCO) (Fig. 3) showed a clear separation of *Vibrio*-treated and untreated samples, and indicated four distinct groupings.

**Table 1:** Results from the multivariate permutational analysis of variance (PERMANOVA) for the effect of time and treatment on bacterial assemblages in the sponge *Aplysilla rosea*.

Source	df	SS	Pseudo-F	P
Time	2	2188	3.654	**0.005**
Treatment	1	3814	6.80	**<0.001**
Tank (Treatment)	4	2370	1.92	0.13
Time x Treatment	1	398	1.29	0.31
Residual	5	1543

Samples from replicate tank 1 for sponge only control and *Vibrio* treated clustered separately from replicates 2 and 3 (Fig. 3). C24-1 and C48-1 were distinguished by a high abundance of *Sinorhizobium* spp. whereas C24-2, C48-2 and C48-3 had high abundances of *Synechococcus* spp., *Oleispira* spp., and *Pirellula* spp. Sample T48-1 was very different from the other replicates (T48-2 and T48-3), being dominated by *Mesorhizobium* spp., *Paracoccus* spp., *Oceanisphaera* spp., *Amphritea balenae* and *Colwellia* spp. (Fig. 2). Sample T24-1 was dominated by *Colwellia* spp., *Ruegeria* spp., *Phaeobacter* spp., *Roseobacter* spp., *Shewanella* spp., *Flavobacterium* spp., *Tenacibaculum* spp, *Arcobacter* spp., and *Pseudovibrio* spp. (in contrast to T24-2 and T24-3, Fig. 2).

The largest differences between treatments at different times were noted for *Synechococcus* spp., which made up 92% of sequences in sponge tissue at the start of the experiment, with a significant interaction (ANOVA: *F*_1,8_ = 6.12 *P* = 0.038) between time and *Vibrio* addition. In the control sponges, *Synechococcus* spp. declined to 60% and 15% of their original abundance after 24 and 48 hours, respectively, and in the *Vibrio*-treated sponges to 15% and 6%, respectively. There was also a significant interaction between time and *Vibrio* treatment on the abundance of *Sinorhizobium* spp. (*F*_1,8_ = 9.42, *P* = 0.015), which increased from 13% at 24 hours for both treated and control samples to 21% in *Vibrio*-treated samples and 80% in control samples. *Vibrio* addition also resulted in significant increases in a number of other taxa (*P* < 0.05) as a percentage of the community (Table 2). Finally, time had an effect for *Phaeobacter* spp., which increased from 0.26% to 0.9% (*F*_1,8_ = 5.62, *P* = 0.045) of the microbial community from 24 to 48 hours.

**Table 2:** Main effect of *Vibrio* addition on relative abundance of microbial 16S rRNA gene sequences in sponges for microbial groups with a significant difference (*P* < 0.05) between control (sponge only) and *Vibrio* treatment. Results from two way ANOVA fitting *Vibrio* addition (Control, +*Vibrio*) x time (24 h, 48 hr), n = 3. There was no significant effect of time for these groups so data from 24 and 48 hours were pooled (n = 6).

Microbial group	% of sequences		ANOVA results
	Control	+*Vibrio*	
*Alteromonas* spp.	0.08	2.21	F_1,8_ = 8.77, *P* = 0.018
*Arcobacter* spp.	0.41	9.09	F_1,8_ = 9.26, *P* = 0.016
*Colwellia* spp.	0.58	3.96	F_1,8_ = 12.45, *P* = 0.008
*C*. *psychrerythraea*	0.66	22.25	F_1,8_ = 40.70, *P* = < 0.001
*Marinomonas* spp.	0.01	0.41	F_1,8_ = 7.50, *P* = 0.026
*Moritella abyssi*	0	1.02	F_1,8_ = 15.18, *P* = 0.005
*Pseudoalteromonas* spp.	0.23	2.07	F_1,8_ = 14.32, *P* = 0.005
*Shewanella* spp.	0.01	0.98	F_1,8_ = 31.67, *P* = < 0.001
*Vibrio natriegens*	0.03	4.08	F_1,8_ = 49.30, *P* = 0.002
*Vibrio* spp.	0.57	7.65	F_1,8_ = 14.32, *P* = 0.005

### Changes in metabolite profiles of sponge extracts

HPLC analysis of metabolites revealed different peak profiles between the control and treated sponges after 48 hours. More peaks were detected at 215 nm wavelength. Partial least squares-discriminant analysis (PLS-DA) of sponge explant HPLC profiles (n = 3) at 215 nm showed distinct clustering patterns after 48 hours between the control and treated sponge extracts (Supplementary File C).

### Bioactivity evaluation of control and treated sponge extracts demonstrated by antibacterial activity and bioautogram

**Figure 4:**
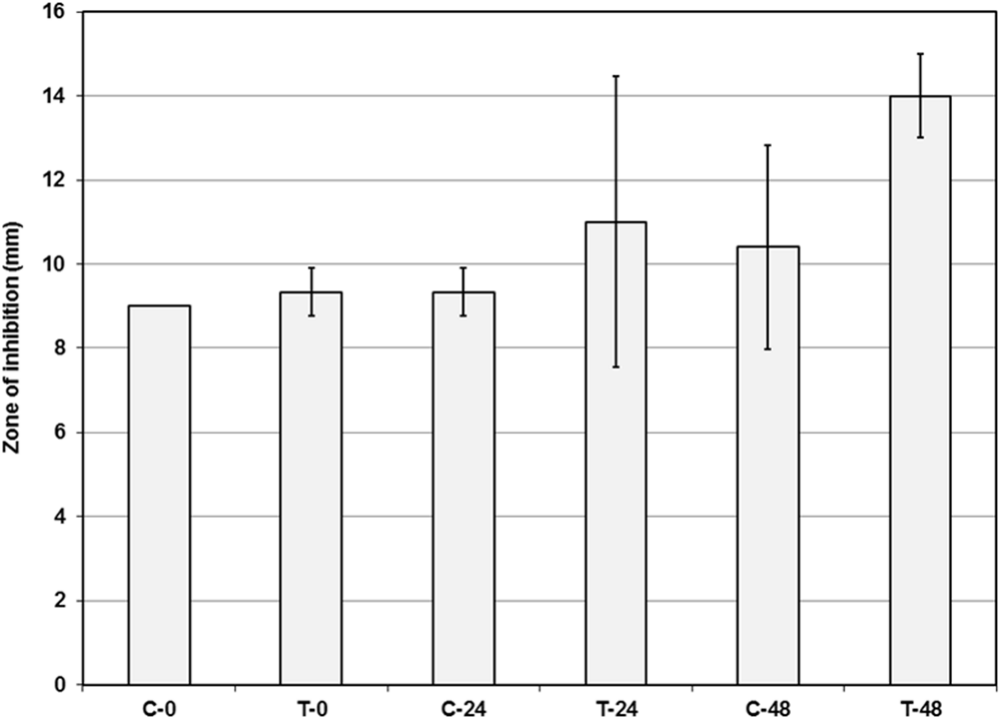
Antibacterial activity of sponge extracts of control and treated tanks against *Staphylococcus aureus* over 48 hours (n = 3 biological replicates, error bar = SD).

To demonstrate changes in bioactivity, sponge extracts from treatment, control tanks and also pure methanol were tested against three selected pathogenic bacteria. The triplicate biological samples at 0 hour showed similar inhibition zones (9 mm) against *Staphylococcus aureus* (Supplementary File D), but after 24 hours the sponge extracts from treated tanks visually showed slightly increased inhibition against *S*. *aureus* than those from control tanks, although this was not statistically significant. This trend remained after 48 hours (Fig. 4). No antibacterial activity was observed from pure methanol against the three selected pathogenic bacteria. The sponge extracts evaluated against *Escherichia coli* and *Vibrio natriegens* also didn’t show any antibacterial activity.

Further evaluation of different spots from the preparative TLC plates against *S*. *aureus* showed that T24-1, T48-1 and T48-2 contained a few active spots (supplementary file E). A Welch two sample t-test indicated that there was no statistically significant difference in antibacterial activity between treated and control samples at 0, 24 or 48 hours.

On the TLC plate a good resolution of compounds was obtained in both Ethyl acetate: Methanol [7:3] and Butanol: Acetic acid: water [4:1:1] (Supplementary File E). When the retention factors between the treatments (T24-1 and T48-2) and the controls (C24-1 and C48-2), were compared, there were more active bands in the extracts of treated sponges compared to extracts of the control sponges, including antibacterial activity against *S*. *aureus* (Supplementary File E), confirmed by Bioautogram (Supplementary File D).

## Discussion

In the marine environment, sponges constantly interact with bacteria within and external to them^27,45^. As filter feeders, sponges filter out many different types of microbes from the surrounding water that may be retained within the sponge either extracellularly or intracellularly^18^. To our knowledge, few experimental studies have been conducted to understand the effect of this interaction on sponge microbial diversity, metabolism and bioactivity^46^. Sponges maintain their natural activity against pathogenic bacteria, viruses, parasites, fungus and other predators by applying their natural products as chemical defence mechanisms^13,47^.

In the present study, the treated sponges appeared to be unhealthy after 48 hours, which could eventually lead to their death (as observed after 54 hours in the pilot study). This decline in health is perhaps due to the accumulation of certain toxic secreted metabolites, as a result of the challenge by a high concentration of *Vibrio natriegens*. In this study, the released metabolites accumulated in the confined system were not captured or removed by any absorbent, which was a shortcoming for this experiment. Further work is needed to decouple the influence of metabolites released by the sponge from that of increased densities of bacteria.

Previous studies confirmed that sponges are able to differentiate between food bacteria that enter the sponge and their own symbionts during feeding^4,48^, which could be the result of discrimination involving the host immune system or by the symbionts’ own mechanism^5^. The change in sponge-associated bacterial diversity, when challenged by either associated or external bacteria, could initiate changes in sponge metabolism and metabolite composition^22,49^. It was not previously known how rapidly the sponge-associated microbial community could change in response to bacterial exposure. We demonstrated, using pyrosequencing, that this microbial community change can occur within 24 hours (Fig. 2). Further support for this result is provided by TRFLP analysis (see Supplementary File F). While the sponge-associated microbial community in the controls also changed over time, perhaps due to a lack of food, there was a vastly different community composition in the sponges treated with *V*. *natriegens* (Fig. 2). In our previous study, a similar phenomenon was also observed when the same sponge species (*Aplysilla rosea*) was exposed to *Streptomyces* ACT-52A^22^.

In the present study, the control sponges were dominated by *Synechococcus* spp. (97%) at the start of the experiment, *Cyanobacteria* (41%, mainly *Synechococcus* spp.) after 24 hours, and *Alphaproteobacteria* (79%, mainly *Sinorhizobium* sp) after 48 hours. It has been reported that *Synechococcus* spp. (“*Candidatus Synechococcus spongiarum*”) is the most common cyanobacterial symbiont in marine sponges^5,50^, although it is very different from free-living *Synechococcus*^51^. Cyanobacteria are one of the most dominant sponge-associated microbial taxa, and perform essential roles in photosynthesis, nitrogen fixation, UV protection, and defensive toxin production^1,52^. However, the functional properties of *Synechococcus* sp. and its symbiotic interaction with sponges remain uncertain^53^ and warrant further study. Moreover, in a recent study it was proposed that genetically distinct clades of “*Candidatus Synechococcus spongiarum*” photosymbionts diverge in their productivity and capable to assimilate carbon and transfer it to the host sponge^54,55^. Our previous study also reported a high prevalence of *Synechococcus*/*Prochlorococcus*, *Pseudovibrio* and *Ruegeria* in *Aplysilla rosea*^22^. *Synechococcus*/*Prochlorococcus* are photosynthetic Cyanobacteria reported in *A*. *rosea* in the past^56^.

Sponges can alter their bacterial assemblage for survival in different environmental conditions^52^, although the environment can also directly alter the bacterial assemblage^57^. In the *Vibrio* treated tanks, the sponge-associated microbial community demonstrated a significant change at both 24 and 48 hours, with a highly diverse microbial structure (Fig. 3). The exposure to *V*. *natriegens* initiated colonization of many minor groups of bacteria, with many different genera detected in the treated sponges at 24 and 48 hours, which were not evident in the controls.

In our previous study it was found that the altered bacterial communities in the treated sponges were dominated by Gamma-proteobacteria (50%) after 24 and 48 hours, but with a different bacterial composition^22^. In contrast, in the present study, after 24 hours *Colwellia* spp., *Vibrio* spp., *Ruegeria* spp. and *Roseobacter* spp. were the main genera and after 48 hours this changed to *Colwellia* spp., *Vibrio* spp. and *Pseudoalteromonas* spp. At both time points, *Colwellia* was one of the major genera found in the treated sponges. *Colwellia* species have their own characteristics and functions in relation to their role in interacting with sponges^58^, however, the reason for the abundance of these genera in the present study require further investigation. Vibrionales of the Proteobacteria, such as *Pseudoalteromonas* and *Vibrio*, have been recognised as the governing antibiotics producers^59,60^. Interestingly, members of the Vibrionales and Alteromonadales may be good probiotics that are active against pathogenic *Vibrio* spp.^61^. A number of exocellular cyclic dipeptides were reported from the *Ruegeria* strain isolated from the sponge *Suberites domuncula*^62^, which may have some role to play in sponge-bacteria interactions.

Exposure time had an effect on the abundance of *Phaeobacter* spp. and two genera from the *Roseobacter* clade; i.e., *Phaeobacter* and *Ruegeria*, which were reported as promising probiotics for marine larviculture^63^. The probiotic (antibacterial) effects of *Phaeobacter* and *Ruegeria* are predominantly caused by their production of the dual-sulfur tropone-derived compound tropodithietic acid (TDA)^63,64^. TDA-producing *Phaeobacter* and *Ruegeria* strains can prevent vibriosis in cod larvae^63^. However, the role of *Phaeobacter* in sponges is still not clear and requires further study.

Moreover, *Colwellia*, *Ruegeria* and *Roseobacteria* spp. are known for the production of bioactive compounds^65^ that may serve as a defence response to *Vibrio* exposure. In contrast, in our previous study^22^, the abundance of cyanobacteria (*Prochlorococcus*), which play a role in carbon metabolism, photosynthesis and nutrition^66,67^, was increased in both control and treated sponges after 48 hours.

It is noted that *V*. *natriegens* grew rapidly in the seawater (Fig. 1), and reached its maximum cell concentration in the control tanks without any sponge explants within 12 hours, revealing a very high CFU/ml compared to *Vibrio anguillarum* in a previous study^3^. This is perhaps due to the fast growing nature of *Vibrio natriegens* in a suitable environment without filtration. On the other hand *Vibrio anguillarum* is known to be slower growing^68^. When *V*. *natriegens* was added to the treatment tanks, the cell concentration decreased rapidly over the first 12 hours, likely due to the rate of filter feeding activity by the sponges^69^, which exceeded the growth rate of *V*. *natriegens*. After 12 hours, *V*. *natriegens* started to increase, and it reached a maximum cell concentration at 24 hours (Fig. 1). This may reflect that the filtering capacity of the sponge decreased due to saturation, or changes in sponge health status due to the toxic compounds released prior to visual deterioration, which was noticeable at 54 hours. A similar trend was observed previously, where overgrowth of *Vibrio* sp. caused significant changes to, and eventually death of, *Hymeniacidon perleve*^70^. In our experiment, the sponges were in a stressed condition in the treated tank at the end of experiments due to the overgrowth of *Vibrio natriegens*. This could occur as *V*. *natriegens* reproduced at a higher rate than grazing by the sponges^3^ or due to the release of toxic metabolites from the sponge body or *Vibrio* fouling the water^3^. Several studies on the cultivation of sponges in aquarium conditions have shown that the morphological change is one of the most important and easiest indicators to monitor sponge health^71^. For this study, sponge morphology was observed visually by checking sponge tissue intactness and colour with photos taken at different time points. Visual observation also confirmed that treated sponges showed a decline in mass during the later stage of the experiment (Supplementary File B). A previous study on the sponge *Aplysina cauliformis* confirmed that diseased sponges were evidenced by a decline of sponge mass^72^. These stressed conditions led to sponge death, similar to the phenomenon observed in this experiment. In contrast, in our previous study it was revealed that the sponge is less vulnerable to a symbiont bacterium even at higher concentrations^22^.

Another impact of the sponge-bacteria interaction could be the notable changes in the biological activities of sponge-derived extracts which evident in a previous study^22^. In contrast, in the present study, initially TLC of treated sponge samples showed interesting result of increasing metabolite bands (Supplementary File E). Later on this change of bioactivity was evaluated by antibacterial activity against *Staphylococcus aureus*, *Escherichia coli* and *Vibrio natriegens*. The result suggested (Fig. 4) (Supplementary File D) increased inhibition of *S*. *aureus* by metabolites from treated samples particularly after 48 hours, although it was not statistically significant. It could be hypothesized that this observed higher bioactivity is related to the colonization of certain bacteria such as *Vibrio* spp. and *Pseudoalteromonas* spp. and their abundance in the sponge^59,60^. Quorum sensing (QS) signals have been detected in *Roseobacter* species^73^, and the production of antibacterial compounds in some bacteria is controlled by QS^74^. It has been suggested as a regulatory mechanism for *Roseobacter* to produce antibacterial compounds^75^. However, the influence of symbiont structure and function on sponge ecology is not well understood in the sponge microbiota and warrants further study^76^.

The current experiment did not include capturing the metabolites released into the sea water by the sponge. Here the system can be improved by introducing absorbents such as XAD7 or XAD4 in mesh bags into the tanks. We applied this approach in a previous study and observed that the metabolites collected from the absorbents showed higher bioactivity compared to sponge tissue^22^. In fact, a continuous flow-through system is desirable. As shown in Supplementary File E, many compounds were produced during the treatment. Further studies can be directed to elucidate these metabolites by purification^77^ and using LC-MS and NMR^78^, which would reveal the metabolite structures.

As it is not easy to observe and test the interaction between sponges and microbes *in situ*, this controlled experimental aquarium system could be a very useful tool to simulate a range of sponge-bacteria interaction conditions where experimental data can be obtained and analysed to test many hypotheses on the roles of the sponge-bacteria interactions. The current approach to the study of sponge-bacteria interactions using a controlled aquarium system does, however, possesses some limitations. In future, the methodologies need to be improved in the experimental system design and also by applying a range of advanced analytical tools including omics tools (genomics, transcriptomics, metabolomics and functional bioassays). Fundamental insights need to be gained, such as the shared metabolic pathways of the sponge and microbiome, the potential role of the microbiome in environmental adaptation of the holobiont^5^, and the scientifically intriguing questions of sponge-bacteria interactions and their roles in biosynthetic metabolism of a myriad of marine natural products, their ecological functions and evolutionary development over 680 million years^79^.

## Conclusions

Through this study we successfully demonstrated the use of a controlled experimental aquarium system to study the role of sponge-bacteria interactions using a sponge *A*. *rosea* and a foreign external bacterium *V*. *natriegens* as a model. Dynamic and concerted responses in microbial diversity, sponge morphology and bioactivity of the sponge were evident. The rapid responses (within 24 h) of sponge microbial diversity and metabolite profile indicate that the sponge is highly responsive to foreign or external bacteria; however over-exposure with high concentrations of foreign bacteria can destroy sponges if released toxicants are not removed and the foreign bacteria are fouling the sponge. The plasticity of sponges when interacting with bacteria within their body and in surrounding seawater makes it an impressive mechanism for adapting sponge microbial diversity. The significance of this controlled aquarium approach to study the sponge-bacteria interactions with a foreign bacterium is its possibility in a range of studies of sponge-bacteria interactions. Further studies should focus on improvements of the experimental system design. Systematic studies should be conducted using different host sponges and their associated bacteria or foreign bacteria to fully elucidate the underlying mechanisms of different sponge-microbe interactions and their adaptation to ecological challenges.

## Electronic supplementary material

Supplementary File

Dataset 1

Dataset 2

Dataset 3
