# Supplementary material for: A controlled aquarium system and approach to study the role of sponge-bacteria interactions using Aplysilla rosea and Vibrio natriegens
Source: Sci Rep. 2018 Aug 7;8:11801. doi: 10.1038/s41598-018-30295-y (PMC6081443; doi:10.1038/s41598-018-30295-y)
Supplement: Supplementary file 1 — Supplementary File [file 41598_2018_30295_MOESM1_ESM.docx]

**A controlled aquarium system and approach to study the role of sponge**-**bacteria** **interactions using *Aplysilla rosea* and *Vibrio natriegens***

Mohammad F. Mehbub^1, 2^*, Jason E. Tanner^1, 3^, Stephen J. Barnett^2^, Jan Bekker^1, 2^, Christopher MM Franco^1, 2^*, Wei Zhang^1, 2^ *

^1^Centre for Marine Bioproducts Development, College of Medicine and Public Health, Flinders University, Bedford Park, SA 5042, Adelaide, Australia.

^2^ Medical Biotechnology, College of Medicine and Public Health, Flinders University,

Bedford Park, SA 5042, Adelaide, Australia.

^3^SARDI Aquatic Sciences, 2 Hamra Avenue, West Beach, SA 5024, Adelaide, Australia.

* Corresponding authors. Tel: +61 8 72218554; fax: +61 8 7221 8555; e-mail: chris.franco@flinders.edu.au (C. Franco), Tel: +61 8 7221 8557; fax: +61 8 7221 8555; e-mail: wei.zhang@flinders.edu.au (W. Zhang), etu.mehbub@flinders.edu.au

**Supplementary File A.** Accession numbers of 16S rRNA genes samples amplified for 454 pyrosequencing with downloadable link.

16S r[RNA-seq of Sponge: bacteria: Sample Treatment.48.3](http://www.ncbi.nlm.nih.gov/sra/SRX950240%5baccn%5d)

1 LS454 (454 GS FLX Titanium) run: 4,253 spots, 2.3M bases, 4.8Mb downloads

Accession: SRX950240

16S r[RNA-seq of Sponge: bacteria: Sample Treatment.48.2](http://www.ncbi.nlm.nih.gov/sra/SRX950239%5baccn%5d)

1 LS454 (454 GS FLX Titanium) run: 4,659 spots, 2.5M bases, 5.2Mb downloads

Accession: SRX950239

16S r[RNA-seq of Sponge: bacteria: Sample Control.48.3](http://www.ncbi.nlm.nih.gov/sra/SRX950238%5baccn%5d)

1 LS454 (454 GS FLX Titanium) run: 11,766 spots, 6.2M bases, 13.1Mb downloads

Accession: SRX950238

16S r[RNA-seq of Sponge: bacteria: Sample Control.48.2](http://www.ncbi.nlm.nih.gov/sra/SRX950237%5baccn%5d)

1 LS454 (454 GS FLX Titanium) run: 10,220 spots, 5.4M bases, 11.4Mb downloads

Accession: SRX950237

16S r[RNA-seq of Sponge: bacteria: Sample Treatment.24.3](http://www.ncbi.nlm.nih.gov/sra/SRX950236%5baccn%5d)

1 LS454 (454 GS FLX Titanium) run: 7,872 spots, 4.2M bases, 8.9Mb downloads

Accession: SRX950236

16S r[RNA-seq of Sponge: bacteria: Sample Treatment.24.2](http://www.ncbi.nlm.nih.gov/sra/SRX950235%5baccn%5d)

1 LS454 (454 GS FLX Titanium) run: 21,798 spots, 11.7M bases, 24.5Mb downloads

Accession: SRX950235

16S r[RNA-seq of Sponge: bacteria: Sample Control.24.2](http://www.ncbi.nlm.nih.gov/sra/SRX950234%5baccn%5d)

1 LS454 (454 GS FLX Titanium) run: 2,340 spots, 1.2M bases, 2.6Mb downloads

Accession: SRX950234

16S r[RNA-seq of Sponge: bacteria: Sample Control.R.0](http://www.ncbi.nlm.nih.gov/sra/SRX950233%5baccn%5d)

1 LS454 (454 GS FLX Titanium) run: 6,300 spots, 3.3M bases, 6.9Mb downloads

Accession: SRX950233

16S r[RNA-seq of Sponge: bacteria: Sample Control.0](http://www.ncbi.nlm.nih.gov/sra/SRX950232%5baccn%5d)

1 LS454 (454 GS FLX Titanium) run: 10,079 spots, 5.4M bases, 11.4Mb downloads

Accession: SRX950232

16S r[RNA-seq of Sponge: bacteria: Sample Control.24.3](http://www.ncbi.nlm.nih.gov/sra/SRX950231%5baccn%5d)

1 LS454 (454 GS FLX Titanium) run: 10,301 spots, 5.4M bases, 11.4Mb downloads

Accession: SRX950231

16S r[RNA-seq of Sponge: bacteria: Sample Control.48.1](http://www.ncbi.nlm.nih.gov/sra/SRX950230%5baccn%5d)

1 LS454 (454 GS FLX Titanium) run: 4,300 spots, 2.3M bases, 5.2Mb downloads

Accession: SRX950230

16S r[RNA-seq of Sponge: bacteria: Sample Treatment.48.1](http://www.ncbi.nlm.nih.gov/sra/SRX950229%5baccn%5d)

1 LS454 (454 GS FLX Titanium) run: 15,858 spots, 8.7M bases, 19Mb downloads

Accession: SRX950229

16S r[RNA-seq of Sponge: bacteria: Sample Control.24.1](http://www.ncbi.nlm.nih.gov/sra/SRX950228%5baccn%5d)

1 LS454 (454 GS FLX Titanium) run: 4,538 spots, 2.4M bases, 5.3Mb downloads

Accession: SRX950228

16S r[RNA-seq of Sponge: bacteria: Sample Treatment.24.1](http://www.ncbi.nlm.nih.gov/sra/SRX950227%5baccn%5d)

1 LS454 (454 GS FLX Titanium) run: 5,529 spots, 2.9M bases, 6.4Mb downloads

Accession: SRX950227

**
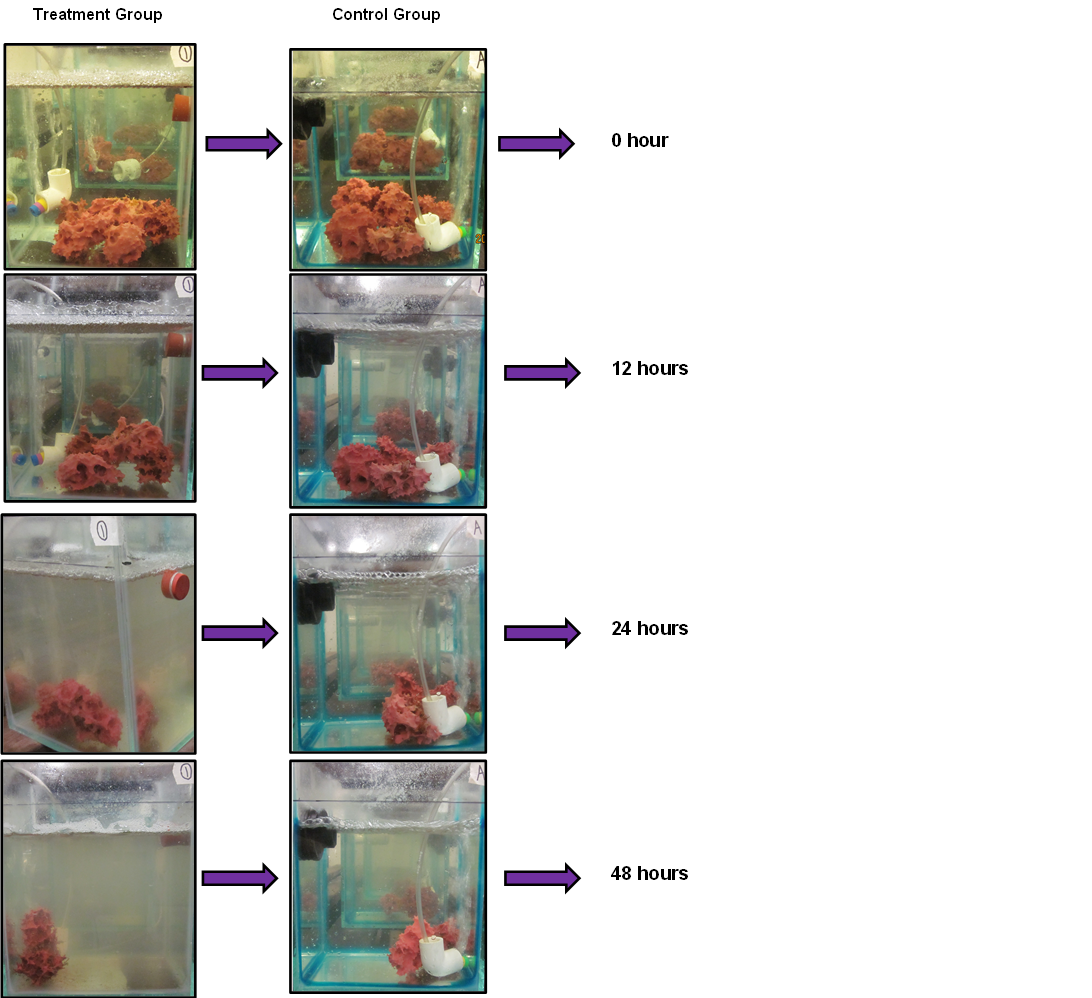
Supplementary File B.** Sponge explants with and without added *Vibrio* at different time points as well as close view of sponge explants after 54 hours.

Legend: Experimental aquarium containing *Aplysilla rosea* sponge explants with added *Vibrio natriegens* (treatment group) and without *V. natriegens* (control group) at different time points.

**
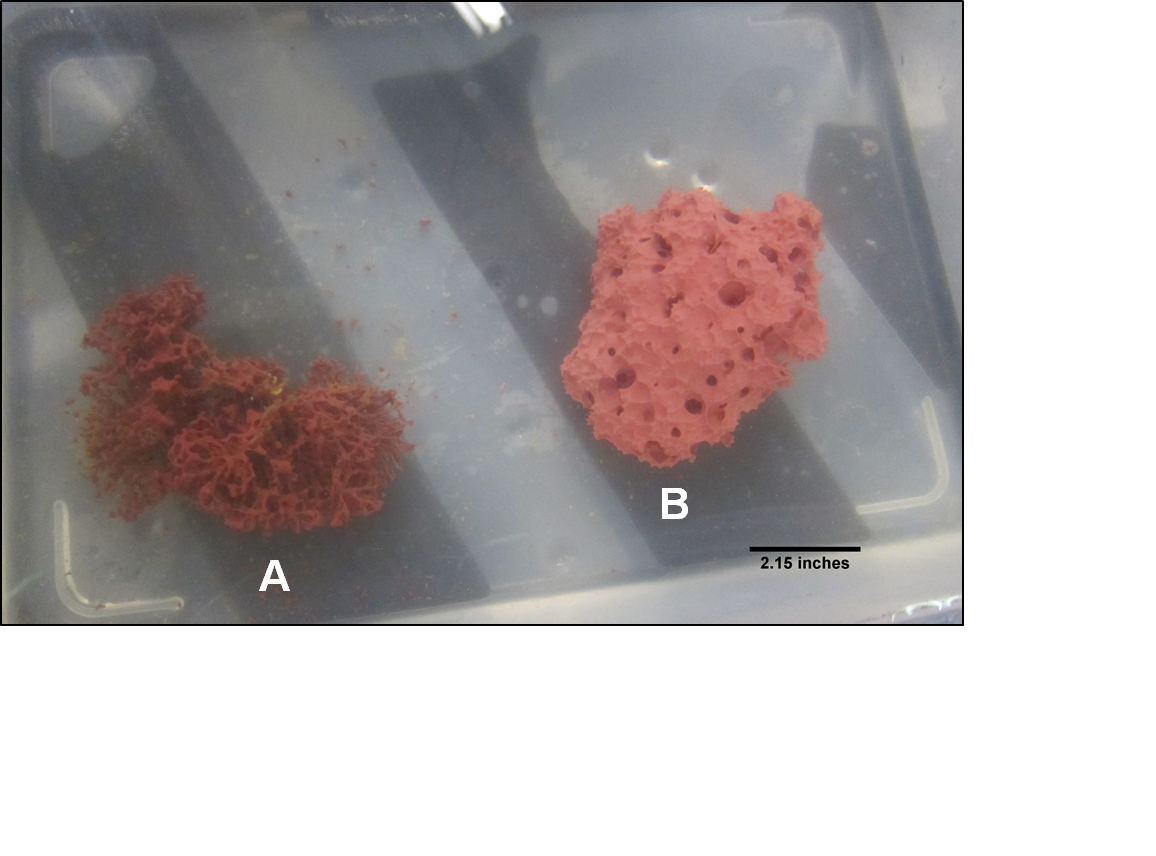
**

**
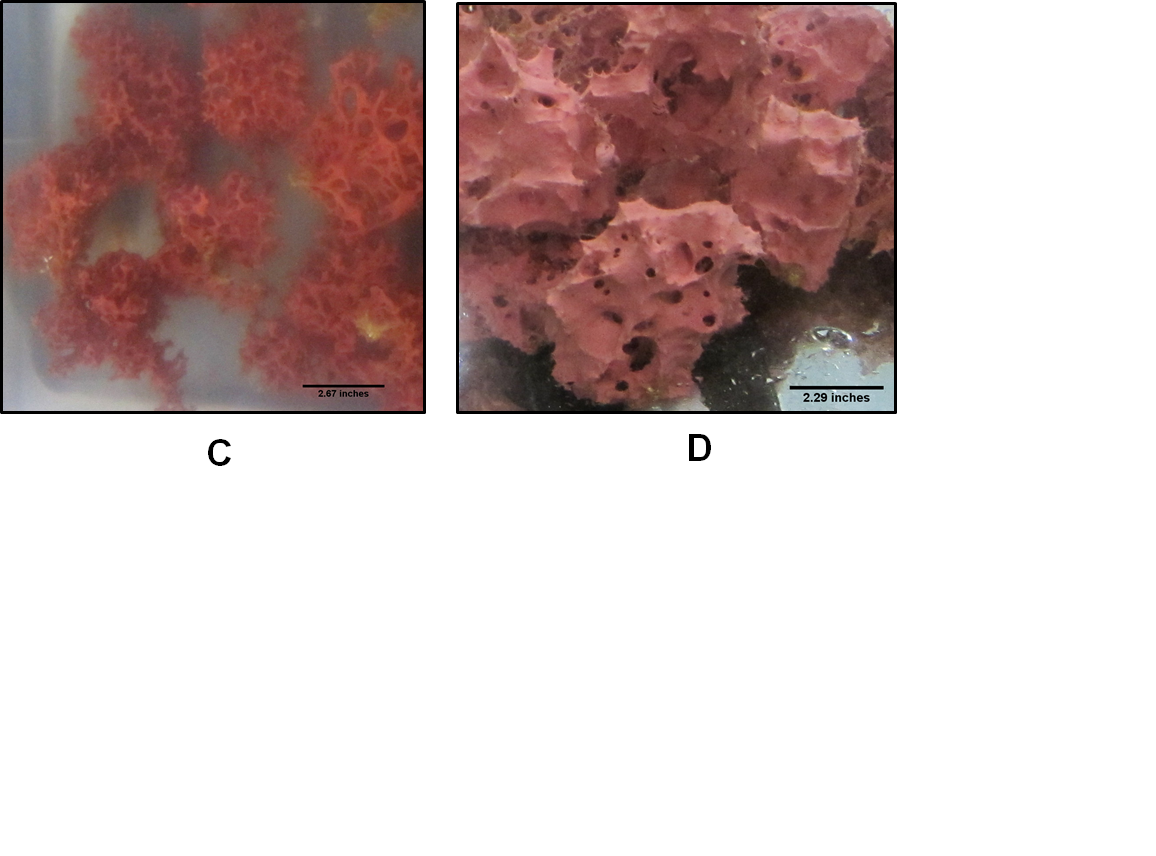
**Legend: **A=** treated and **B=** control sponge explants after 54 hours.

Legend: **C=** treated (9 replicates) and **D=** control (9 replicates) sponge explants from pilot experiment after 54 hours.

**Supplementary File C.** Partial least squares-discriminant analysis of polar metabolites of the sponge *Aplysilla rosea* analysed by HPLC between control (C) and treatment tanks (T) at 0, 24 and 48 h for each replicate tank (n=3) at 215 nm wavelength.

**
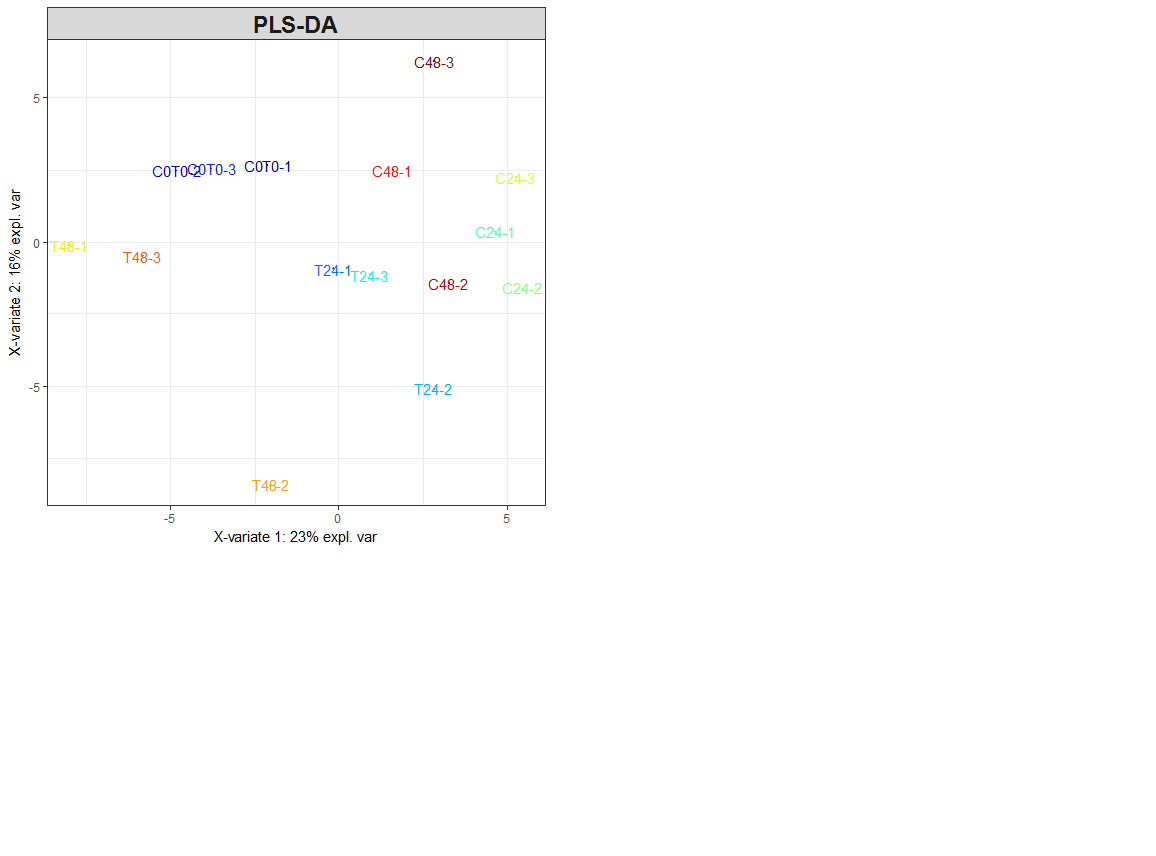
**

Figure Legends: C0T0-1, C0T0-2, C0T0-3= sponge extracts at 0 hour both for control and treatment; T24-1, T24-2, T24-3= Treated sponge extracts at 24 hours; C24-1, C24-2, C24-3= Control sponge extracts at 24 hours; T48-1, T48-2, T48-3= Treated sponge extracts at 48 hours; C48-1, C48-2, C48-3= Control sponge extracts at 48 hours.

**Supplementary File D**. Antibacterial activity and bioautogram of sponge extracts from control and treated samples against *Staphylococcus aureus* after 0, 24 and 48 hours.

**
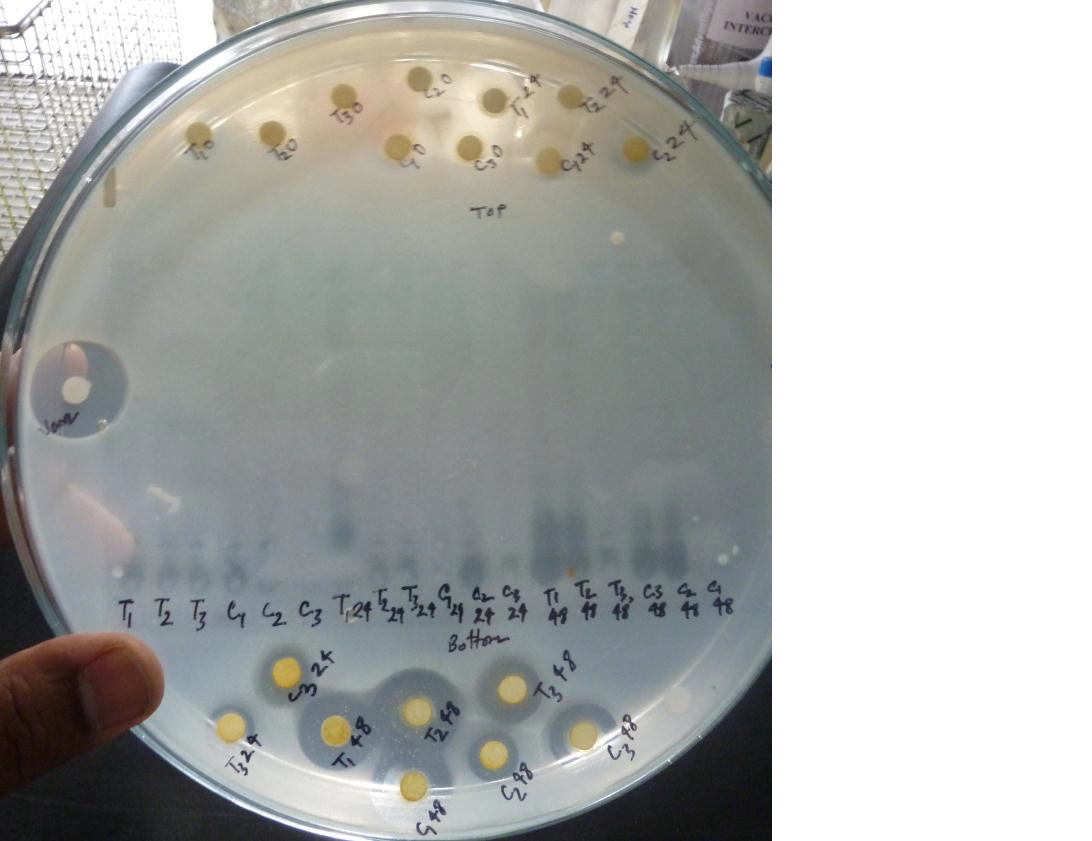
**

Figure Legends: T_1_, T_2_, T_3_= Treated sponge extracts at 0 hour; C_1_, C_2_, C_3_= Control sponge extracts at 0 hour; T_1_24, T_2_24, T_3_24= Treated sponge extracts at 24 hours; C_1_24, C_2_24, C_3_24= Control sponge extracts at 24 hours; T_1_48, T_2_48, T_3_48= Treated sponge extracts at 48 hours; C_1_48, C_2_48, C_3_48= Control sponge extracts at 48 hours; Van= Vancomycin.

**Supplementary File E**. Retention factors of sponge extract with Ethylacetate 7: Methanol 3 and Butanol 4; Acetic acid 1: Water 1, solvent system and their TLC profile at 365 nm and at 254 nm respectively.

| **Name of Sample** | **Spot** | **Retention Factor** | |
| --- | --- | --- | --- |
|  |  | **E:M=7:3 (365 nm)** | **B:A:W=4:1:1 (254 nm)** |
| **T0-1 and C0-1** | 1 | 0.13 | 0.15 |
|  | 2 | 0.62 | 0.4 |
|  | 3 | 0.75 | 0.53 |
|  | 4 | - | 0.66 |
|  | 5 | - | 0.85 |
|  | 6 | - | 0.88 |
|  |  |  |  |
| **T24-1** | 1 | 0.09 | **0.28** |
|  | 2 | **0.19** | **0.37** |
|  | 3 | **0.38** | 0.41 |
|  | 4 | **0.53** | **0.48** |
|  | 5 | **0.60** | 0.56 |
|  | 6 | 0.69 | **0.80** |
|  | 7 | **0.74** | - |
|  | 8 | 0.82 | - |
|  |  |  |  |
| **C24-1** | 1 | 0.09 | 0.23 |
|  | 2 | 0.17 | 0.31 |
|  | 3 | 0.34 | 0.41 |
|  | 4 | 0.69 | 0.52 |
|  | 5 | 0.75 | 0.56 |
|  | 6 | 0.82 | 0.83 |
|  |  |  |  |
| **T48-2** | 1 | **0.09** | **0.30** |
|  | 2 | 0.19 | **0.38** |
|  | 3 | **0.22** | 0.41 |
|  | 4 | **0.39** | **0.47** |
|  | 5 | **0.51** | **0.52** |
|  | 6 | **0.67** | **0.60** |
|  | 7 | **0.72** | **0.78** |
|  | 8 | **0.75** | - |
|  |  |  |  |
| **C48-2** | 1 | 0.11 | 0.23 |
|  | 2 | 0.19 | 0.32 |
|  | 3 | 0.35 | 0.41 |
|  | 4 | 0.71 | 0.50 |
|  | 5 | 0.76 | 0.53 |
|  | 6 | 0.85 | 0.65 |
|  | 7 | - | 0.83 |
|  | 8 | - | 0.88 |


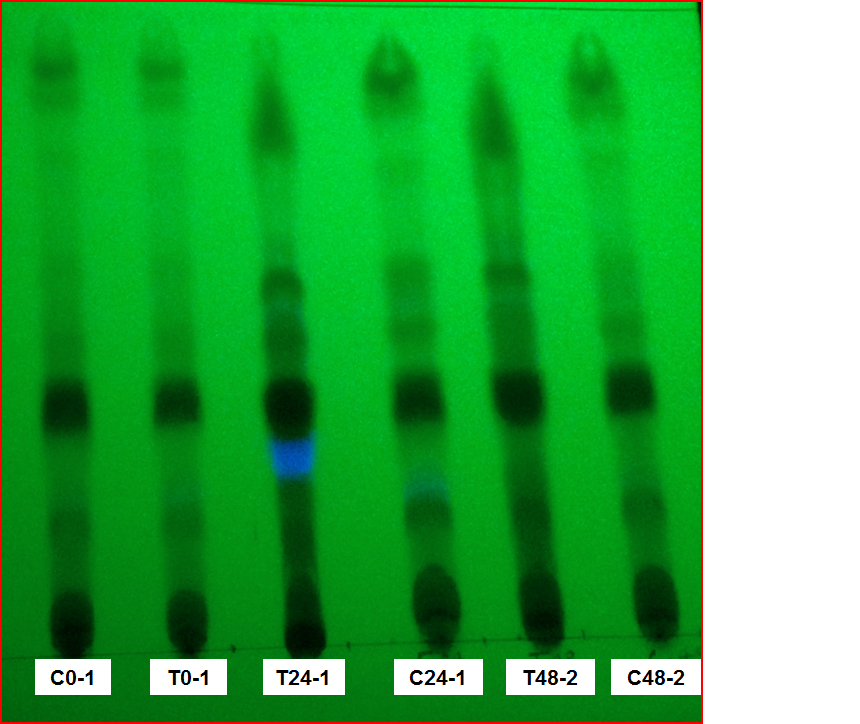

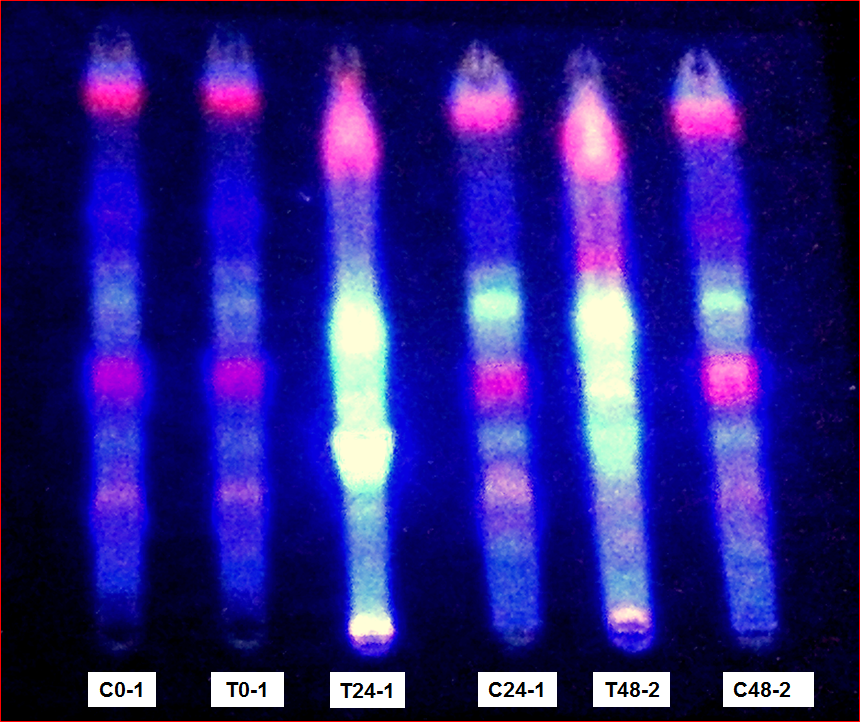


Butanol 4; Acetic acid 1: Water 1 at 254 nm

Ethylacetate 7: Methanol 3 at 365 nm

Legends: C0-1= Control at 0 hour replicate 1, T0-1= Treatment at 0 hour replicate 1; C24-1=Control at 24 hours, replicate 1, T24-1=Treatment at 24 hours replicate 1; C48-2=Control at 48 hour replicate 2, T48-2=Treatment at 48 hours replicate 2.

**Supplementary File F.** TRFL-P method and details result

**TRFL**-**P analyses**

Microbial community analysis by TRFL-P was carried out on sponge DNA samples at 0, 24 and 48 hours. Approximately 400 ng of purified PCR products were digested with the restriction 316 endonucleases, Hha1, Msp1 and Rsa1 in a total reaction volume of 50 μl following the manufacturer’s protocol. Immediately following digestion, samples were inactivated by heating 318 at 65°C for 20 min for Hha1 and Rsa1 digested products. Msp1 was inactivated by adding 2.5 μl 319 of 0.5 M EDTA. Samples were sent to Macrogen, Korea for fragment analysis. The company provided the raw FSA files after fragment analysis. Data were normalized using Dunbar’s method^1^. Peak profiles were generated using Peak Scanner Software v 2.0 (Life Technologies), and peaks (putative populations) were identified using Microbial Community Analysis III (MiCA3)^2^ together with the Ribosomal Database Project (RDP) database.

**Table A.** Name of the primers used for TRFL-P study.

| **Name of Primer for TRFL**-**P** | **Sequence** | **Reference** |
| --- | --- | --- |
| 9F | 6 FAM 5^/^ GAGTTTGATCMTGGCTCAGAT 3^/^ | New |
| 928R | 5^/^ CCCCGTCAATTCMTTTGAGT 3^/^ | New |
| EUB 8F | 6 FAM 5^/^ AGAGTTTGATCMTGGCTCAG 3^/^ | ^3^ |
| EUB 926R | VIC 5^/^ CCGTCAATTCMTTTRAGTTT 3^/^ | ^3^ |

**Table B.** Putative genera identified by TRFL-P using primer pairs 9F-928R and EUB 8F-EUB 926R.

| **Sample and Time** | **Primer 9F and 928R** | **Primer EUB 8F and EUB 926R** |
| --- | --- | --- |
|  |  |  |
| Treatment+ Control (T=0h, C=0h) | *Acinetobacter, , Burkholderia, *****Colwellia****, *****Pseudoalteromonas****, *****Pseudomonas****, Ralstonia, *Vibrio, Janthinobacterium,* ***Microbulbifer****, Pusillimonas,* **Unknown** | *Bacillus,* *Bacteroidetes*, *Bradyrhizobium*, ***Candidtaus***, *Clostridium*, *Comamonas*, *Mesorhizobium*, *Rhizobiales*, ***Rhizobium***, *Streptomyces*, ****Synechococcus***, *Streptococcus*, **Vibrio.* Unidentified and unknown |
| Treatment (T=24h) | ***Achromatium***, *Achromobacter*, ***Acidithiobacillus***, ***Acidovorax***, *Aeromonas*, ***Alcaligenes***, *Alicycliphilus*, ***Aliivibrio***, *Arthrobacter*, ***Bacillus***, *Beggiatoa*, ***Buchnera***, ***Candidatus***, ***Chromatium***, **Comamonadaceae**, ***Comamonas***, ***Delftia***, ***Diaphorobacter***, *Enterobacter*, *Escherichia*, *Gallibacterium*, ***Haemophilus***, *Malikia*, ***Neptunomonas***, *Nitrosococcus*, *Olavius*, ***Pasteurella***, *Polaromonas*, *Serratia*, ***Shewanella***, *Shigella*, ***Streptomyces***, *Thioalkalivibrio*, *Uchneara*, ***Variovorax*** | ***Achromatium***, *Acidiphylum*, *Acidithiobacillus*, *Acidovorax*, *Actinobacillus*, *Acinetobacter*, ***Actinomyces***, *Aliivibrio*, **Alteromonas, *Archobacter* , ***Azorhizobium***, *Bacillus*, *Buchnera*, *Candidatus*, ****Colwellia***, *Clostridium*, *Flavobacteria*, **Mesorhizobium* , *Pseudomonas*, *Pseudoalteromonas*, ****Roseobacter***, ****Ruegeria***, **Sinorhizobium*, *Vibrio*, **Synechococcus*, ***Streptomyces***, *Shewanella*, unidentified and unknown |
| Control (C=24h) | *Capnocytophaga*, ***Duganella*,** *Ethylomicrobium*, *Ethylophaga*, ***Ethylophilus***, *Massillia*, ***Psychrobacter*** and *Zoogloea* | Actinobacteria, *Arthrobacter*, *Bacillus*, *Candidatus*, *Clostridium*, ****Pseudomonas***, ****Synechococcus***, *Streptomyces* and ***Rhizobium*** |
| Treatment (T=48h) | ***Acinetobacter***, *Beggitoa*, , *Capnocytophaga*, *Citrobacter*, Comamonadaceae, *Eikenella*, ***Flavobacterium***, *Pasteurella,* Uncultured, **Unidentified** | *Achromatium*, *Achromobacter*, *Acidithiobacillus*, , ***Acinetobacter***, *Actinobacillus*, *Actinomyces, *Alteromonas* , *Arthrobacter*, *Azorhizobium*, *Azospirillum*, *Bacillus*, *Beggiatoa*, *Bordetella*, *Burkholderia*, *Candidatus*, *Capnocyophaga*, *Cellvibrio*, *Clostridium*, ****Colwellia***, *Corynebacterium*, *Deinococcus*, *Desulfovibrio*, ***Flavobacteria****, Halobacillus*, *Lactobacillus*, ***Mesorhizobium***, *Methylobacter*, ***Phaeobacter***, *Pseudoalteromonas*, *Pseudomonas*, *Rhizobium*, *Rhodococcus*, ****Roseobacter***, ****Ruegeria***, *Salnimicrobium*, *Shewanella*, **Sinorhizobium*, *Sphingomonas*, *Streptomyces*, **Synechococcus*, **Vibrio,* Unknown |
| Control (C=48h) | *Achromatium*, ***Candidatus***, *Chromatium* and *Ethylococcus* | *Bacillus*, ***Candidatus***, *Clostridium*, **Flavobacteria*, *Pseudoalteromonas*, *Pseudomonas*, *Rhizobium*, ****Sinorhizobium***, *Streptomyces*, **Synechococcus*. |

- * indicating genera matching with pyrosequencing result

**Bold** indicating most abundant genera.

1 Dunbar, J., Ticknor, L. O. & Kuske, C. R. Phylogenetic specificity and reproducibility and new method for analysis of terminal restriction fragment profiles of 16S rRNA genes from bacterial communities. *Appl. Environ. Microbiol.* **67**, 190-197; Doi:10.1128/AEM.67.1.190-197.2001 (2001).

2 Shyu, C., Soule, T., Bent, S. J., Foster, J. A. & Forney, L. J. MiCA: a web-based tool for the analysis of microbial communities based on terminal-restriction fragment length polymorphisms of 16S and 18S rRNA genes. *Microb. Ecol.* **53**, 562-570; Doi:10.1007/s00248-006-9106-0 (2007).

3 Lane, D. J. in *Nucleic Acid Techniques in Bacterial Systematics* 115-148 (J. Wiley, 1991).
